# Child Poverty and Its Associated Issues in A City, Tokyo: Insights from Community-Based Participatory Research

**DOI:** 10.3390/children12020252

**Published:** 2025-02-19

**Authors:** Shinpei Ikeda, Yuriko Iwabuchi, Masato Nakamura, Kanta Ohno, Hirotomo Shibahashi

**Affiliations:** 1 Major of Occupational Therapy, Department of Rehabilitation, School of Health Sciences, Tokyo University of Technology, Tokyo 144-8535, Japan; ohnoknt@stf.teu.ac.jp (K.O.); shibahashiht@stf.teu.ac.jp (H.S.); 2 Institute for Gerontology, J. F. Oberlin University, Tokyo 194-0294, Japan; 3 Social Welfare Corporation Kikakukai, Tokyo 205-0023, Japan

**Keywords:** child poverty, community-based participatory research (CBPR), qualitative study, Japan

## Abstract

Background/Objectives: In recent years, despite growing awareness of child poverty in Japan, research examining its impact on the daily lives of children and their families within schools and communities remains limited. This study aims to clarify the extent of child poverty and associated issues in A City, Tokyo, through qualitative research conducted as part of the Community-Based Participatory Research (CBPR) process. Methods: Twelve research participants were selected, including Vice Principals of schools, Community and Child Welfare Officers, and other stakeholders involved in supporting economically disadvantaged households raising children. A focus group discussion (FGD) was conducted on 16 October 2021 to examine the current state of child poverty in A City and local initiatives addressing the issue. Data were analyzed using a qualitative descriptive approach. Results: Three categories emerged from the analysis: (1) Children Disadvantaged by Their Family’s Financial Situation, (2) Challenges in Supporting Financially Struggling Families in Schools and Communities, and (3) the Necessity of Establishing Comprehensive Systems to Support Families. These findings highlight the complex challenges schools and communities face in supporting economically disadvantaged families. Conclusions: Addressing child poverty in A City requires strengthening collaboration between the education sector and community stakeholders, enhancing early detection of poverty-related issues, and establishing comprehensive support systems for timely intervention. However, cultural norms in Japan, such as the reluctance to impose a burden on others, might create barriers to seeking assistance. To overcome these challenges, CBPR is expected to play a key role in fostering networks among children, parents, and support providers.

## 1. Introduction

UNICEF’s Innocenti Report Card 18 provides a comparative analysis of child poverty rates among 43 OECD (Organization for Economic Co-operation and Development) and EU (European Union) countries. Japan ranked 11th out of 39 countries, with an average child poverty rate of 14.8% between 2019 and 2021 [1], meaning that approximately one in seven children in Japan live in poverty. A key aspect of child poverty in Japan is relative poverty, often referred to as “invisible poverty”, which signifies a living standard much lower than that of the majority of the population, rather than extreme deprivation. This form of poverty is particularly prevalent among children in Japan, making it difficult for many to recognize despite its significant impact on certain members of society. The roots of child poverty in Japan can be traced back to the collapse of the economic bubble in the 1990s. This event led to a period of slow economic growth, which exacerbated economic disparities. The rise in unstable employment and non-regular workers further contributed to the growing poverty rates, particularly among children. These structural changes in the labor market have had long-lasting effects, increasing the number of families struggling to provide for their children, thus deepening the issue of child poverty.

To contextualize Japan’s situation from a comparative perspective, it is useful to examine South Korea, another developed country in East Asia. South Korea has a child poverty rate of 15.7%, ranking 15th globally [1]. According to the OECD, key factors contributing to child poverty in South Korea include an increase in highly educated fathers engaged in non-regular employment; minimal financial contributions from maternal employment due to low-wage, non-regular jobs; and the high cost of education, which places a heavy financial burden on families. Households cover 42% of education expenses—nearly double the OECD average—and widespread reliance on private tutoring services (“Hakwon”) further exacerbates financial strain [2]. According to Weon S. et al., material deprivation (15%) is more prevalent than income poverty (12%) [3]. Their study suggests that about one in three children in Korea is either poor or at risk of poverty, raising concerns about the adequacy of income-based measures in capturing the full scope of child poverty [3].

In contrast, child poverty in South Asia is characterized by absolute poverty. The United Nations Development Program (UNDP) reports on the reality of child poverty in the region using the Multidimensional Poverty Index (MPI), which assesses poverty through ten indicators across three key dimensions: health, education, and standard of living [4]. In South Asia, which includes Afghanistan, Bangladesh, Bhutan, and India, 22.7% of children under the age of five experience intrahousehold nutritional inequality, meaning that at least one child in the household is malnourished while another is not. This percentage rises to over one-third in Pakistan. Additionally, 36.7 million school-age children across the region do not attend school through grade 8, with 88.0% of them living in multidimensionally poor households. Gender disparities are evident, as 10.7% of girls and 9.0% of boys in South Asia are out of school while living in multidimensionally poor households. The gap is particularly striking in Afghanistan, where 44.0% of girls and 24.8% of boys aged 7–15 face this situation [4]. These results demonstrate that child poverty in South Asia is fundamentally linked to absolute poverty.

While absolute poverty is a critical issue in developing countries, in developed nations like Japan and South Korea, relative poverty remains a pressing concern. According to a review by Wise PH, the long-term impact of childhood poverty on adult health and aging has been firmly established through a substantial body of evidence [5]. This research consistently demonstrates that childhood poverty and early life experiences significantly influence major health risks in adulthood, including cardiovascular diseases, obesity, diabetes, certain cancers, mental health disorders, osteoporosis, fractures, and potentially dementia. Several reports from Asia, including Japan, also support these findings. One study suggests that adult socioeconomic status (SES) and social support together mediate a significant portion of the impact of childhood poverty on adult health risk factors [6]. Specifically, childhood poverty is a reliable predictor of smoking, lack of exercise, and poor dietary habits in later life. Moreover, individuals who experienced childhood poverty are more likely to face educational disadvantages, remain in unstable employment, and receive social support in the lowest tertile in adulthood [6]. These experiences of childhood poverty also have lasting effects on adult self-rated health [7] and subjective well-being [8]. These findings highlight the importance of addressing child poverty in order to prevent the perpetuation of socioeconomic, health, and psychological well-being disparities across generations. In the context of child food security in Japan, children living in poverty are more likely to exhibit poor dietary habits, such as skipping breakfast and consuming fewer vegetables [9]. Additionally, their parents are less likely to engage in positive behaviors, such as discussing school life with them or preparing meals. The COVID-19 (Coronavirus Disease 2019) pandemic further exacerbated this situation by disrupting external food support systems, such as school meals, which had previously served as a safety net for vulnerable children [10]. This disruption highlights the increasing need for community-based support initiatives, such as children’s cafeterias, to address the nutritional needs of children during times of crisis. Furthermore, the pandemic also destabilized parental employment, thereby increasing the financial strain on families [11]. These combined effects deepened the challenges faced by children in poverty, particularly in terms of access to food and educational resources.

To ensure a nonpoor standard of living for families with children, income from work is essential; however, it may be insufficient if there are not enough adults working adequate hours or if wages are too low. Social cash and near-cash transfers, such as child benefits, unemployment benefits, housing benefits, and food subsidies, play a crucial role in alleviating severe poverty. Therefore, both income from work and social transfers are vital for addressing child poverty [12,13]. The Japanese government’s response to child poverty is guided by the General Principles of Policy on Poverty among Children, established in 2014. The government’s priority measures include (a) educational support, (b) support for a stable lifestyle, (c) employment support for parents/guardians, and (d) economic support [14]. These measures are advocated by local municipalities, with an emphasis on schools as central platforms where School Social Workers (SSWs) can connect children and their families to local resources. If community networks were effectively utilized, support for impoverished families could be delivered promptly and flexibly, while also facilitating the early identification of socially isolated children. However, interactions within families, schools, and communities suggest that children might experience exclusion from key opportunities due to stigma, economic constraints, and family stress [15]. Lipina SJ et al. argue that traditional measures of child poverty are inadequate because they overlook age-appropriate developmental needs and the cultural contexts in which children live [16]. They also suggest that failing to consider multifaceted needs, such as emotional support and freedom, may result in overlooking the true needs of children [16]. In response to these issues, research focusing on the experiences of children facing poverty in schools and communities, particularly in the context of Japan, remains absent.

In this context, Social Welfare Corporation B (hereinafter referred to as “Corporation B”) in A City, Tokyo, recognized the need to address child poverty in the local community by understanding its current state and the existing support mechanisms. Therefore, this study aims to clarify the extent of child poverty and the issues associated with it through qualitative research. By examining how child poverty is perceived and managed at the local level, this study highlights gaps within the existing support networks and provides insights into more effective, community-based solutions.

## 2. Materials and Methods

### 2.1. Theoretical Foundation and Participant Selection

This study is based on Community-Based Participatory Research (CBPR), a research approach in which local community members collaborate with professionals and researchers to address health issues within their community. According to the reviews of CBPR research, the processes generally progress through three stages: (a) incorporating community and cultural knowledge into interventions/research, (b) empowering partners to work together well, and (c) involving community members throughout the research [17]. Our study focuses on the early phases of the CBPR process, where stakeholders work together to understand the current state of child poverty in A City and engage in sharing perspectives to build mutual understanding, all while respecting the local community’s culture.

A City, the focus area of this study, is located in the Tama region of Tokyo, primarily consisting of residential and industrial zones. As of 2021, A City’s total population was approximately 54,000, with around 2700 students attending public elementary schools and about 1400 attending public junior high schools. According to the 2021 data from the Family Income and Expenditure Survey by the Statistics Bureau of Japan, among the 47 prefectural capital cities, Gifu City in Gifu Prefecture recorded the highest average monthly household income at approximately JPY 651,000 (approximately USD 5924 at the 2021 average exchange rate of JPY 109.9 per USD). Tokyo’s 23 wards ranked second with an average income of approximately JPY 646,000 (approximately USD 5879), while Kofu City in Yamanashi Prefecture had the lowest income at approximately JPY 364,000 (approximately USD 3311). These figures are based on data limited to workers’ households of total households [18]. According to Hashimoto’s report on class composition by municipality in the Tokyo metropolitan area from 1990 to 2010, the capitalist class was most prevalent in the central and western parts of the 23 wards in 2010, while A City had the lowest proportion. In contrast, the working class was more concentrated in areas where the capitalist class was less dominant [19]. In terms of household income in 2010, the municipality with the highest average annual household income in Tokyo recorded JPY 8.224 million (approximately USD 93,774 at the 2010 average exchange rate of JPY 87.7 per USD), with 7.2% of households earning less than JPY 2 million (approximately USD 22,786) and 23.9% earning more than JPY 10 million (approximately USD 113,892). In comparison, A City’s average household income was JPY 4.894 million (approximately USD 55,740), with 16.2% of households earning less than JPY 2 million and 6.7% earning more than JPY 10 million. Among the 53 wards and municipalities in Tokyo, A City ranked 34th in average household income, while the municipality with the lowest average income recorded JPY 4.03 million (approximately USD 45,904). At the time this study was conducted in 2021, the local government had not yet conducted any surveys on child poverty in A City. As a result, there were no available data on the proportion of children living in poverty or household income in A City.

Located in the southeastern part of A City, Corporation B operates an elderly care facility. Since its establishment, it has closely collaborated with nearby elementary and junior high schools to provide experiential lessons on elderly welfare, strengthening its ties with the community. In addition, Corporation B serves as one of A City’s disaster response hubs, maintaining stockpiles of food, water, and emergency supplies such as divers. At the time of the study, it actively engaged in new initiatives to address child poverty, collaborating with A City’s food bank to expand food assistance and develop further support programs. The research participants consisted of 12 individuals, including Vice Principals of local schools E and F, Community and Child Welfare Officers G and H, and others involved in various organizations and roles. For a detailed description of the participants, refer to Table 1. These participants were selected by the lead researcher and members of Corporation B based on their involvement with economically disadvantaged households raising children. The selection focused on those engaged in A City’s child poverty alleviation efforts, particularly in education, food security, and youth development, including educational programs, youth welfare, and the food bank.

### 2.2. Data Collection and Analytical Approach

A focus group discussion (FGD) was conducted with the research participants. FGD is a qualitative research method where a small group discusses a specific topic. The goal is to gather in-depth insights through group interaction, allowing participants to influence each other’s opinions. Before addressing the main themes of the discussion, the facilitator introduced the current state of relative child poverty in Japan and highlighted the role of schools and teachers as key points of support in addressing the issue, particularly in light of the expectations set by the Japanese government. The FGD addressed two key themes: (1) What is the current state of child poverty in A City? (2) What initiatives are currently being implemented within the local community to address this issue, and what are the challenges they face? Participants were encouraged to freely share their opinions and experiences. The discussion lasted approximately 90 min, and participants’ responses were recorded using an IC recorder. The FGD was held on 16 October 2021.

We adopted a qualitative descriptive research approach for the analysis. This method focuses on capturing a detailed, comprehensive account of the phenomenon, presenting participants’ own words and interpretations of their lived experiences without imposing theoretical frameworks [20]. The analysis began by transcribing the content recorded on the IC recorder. Our study extracted meaningful units related to key themes and assigned codes, which were subsequently grouped into subcategories. The relationships among these subcategories were then examined, culminating in the creation of categories with higher levels of abstraction. The analysis was conducted by the first author and co-authors, who reviewed and refined the methods and descriptions to ensure rigor.

## 3. Results

Three categories emerged from the analysis: Children Disadvantaged by Their Family’s Financial Situation, Challenges in Supporting Financially Struggling Families in Schools and Communities, and the Necessity of Establishing Comprehensive Systems to Support Families. The following sections describe each category, using relevant subcategories and representative codes (Table 2).

### 3.1. Children Disadvantaged by Their Family’s Financial Situation

The FGD reveals that children from financially disadvantaged families face challenges in various areas, including school-related costs and food security. This section outlines how financial difficulties affect children’s access to basic needs like education and nutrition.

#### 3.1.1. Households Unable to Afford School-Related Costs

- “In a class of 30 to 35 students, about 5 of them have difficulty paying for school lunches” (E.).
- “There are households that are behind on payments for things like study materials and school lunches” (F.).

This subcategory highlights the financial strain on families unable to cover basic school-related expenses. Struggling to afford school lunches and study materials limits children’s access to essential educational resources, hindering their ability to fully engage in school.

#### 3.1.2. Students Unable to Participate in School Events Due to Financial Constraints

- “Some students couldn’t participate in school events because they didn’t have the money” (F.).
- “There are a certain number of families who find it hard to save up for the cost of the school trip” (E.).

Financial constraints prevent some children from participating in school events, crucial for their social and educational development. This subcategory reflects how such financial barriers limit their opportunities for extracurricular engagement, like school trips.

#### 3.1.3. Children Who Lack Sufficient Meals at Home

- “I imagine that many children don’t eat breakfast before coming to school” (I.).
- “There was a student who depended on school meals for nutrition—it was a lifeline for them” (F.).

Food insecurity emerged as a major concern, with some children not receiving enough nourishment at home. For one child, school meals are their primary source of nutrition, underscoring the critical role schools play in meeting the needs of low-income families.

### 3.2. Challenges in Supporting Financially Struggling Families in Schools and Communities

Participants discussed the challenges schools and communities face in supporting financially struggling families, such as identifying those in need, the reluctance to seek help, and weakened community networks. This section outlines key subcategories reflecting the complexities of addressing these families’ needs.

#### 3.2.1. Difficulty in Offering Support to Families Struggling with Financial Hardship in Schools

- “Many families aren’t struggling enough to qualify for financial support, like school aid” (G.).
- “Kids don’t speak up and say ‘I’m in trouble’ on their own” (E.).
- “Even if we notice that a student isn’t eating, it’s hard to contact the parents easily” (F.).
- “It’s difficult to understand a student’s family situation—it’s just not easy to find out” (O.).

A key challenge for schools is that many families, despite financial hardship, do not qualify for formal support, like school aid. Students are often reluctant to share their difficulties, making it hard for their teachers to identify them, which limits the effectiveness of several supports.

#### 3.2.2. Difficulty in Gaining Insight into Families with Children Facing Hardships in the Community

- “In one visit to a single mother’s home, when we asked about their financial situation, they refused to answer” (G.).
- “I think parents don’t ask for help because of pride” (L.).
- “I imagine it’s hard for parents to accept free donations or assistance—there’s resistance to that” (J.).
- “Some families refuse to have child welfare services visit, and it’s hard to get involved with them” (H.).

Outside of schools, identifying families’ financial struggles is challenging. Many, especially single-parent households, avoid disclosing their hardships due to pride. This hinders support efforts, with some families even cutting off their own support networks by refusing assistance from child welfare services.

#### 3.2.3. Isolation of Families Due to the Weakening of Neighborhood Communities

- “People just don’t care about what’s going on next door anymore” (K.).
- “The neighborhood association is weakening, and we don’t even know who our neighbors are” (H.).
- “With COVID-19, nuclear families have become more common, and now it’s more of a world centered on individual families” (J.).
- “The way parents live has a direct impact on their children’s environment” (I.).

Weakened neighborhood bonds have led to increased family isolation. Reduced interaction between neighbors limits opportunities for mutual support. Additionally, the decline of local organizations and the rise of nuclear families, particularly post-COVID, has fragmented the sense of community.

### 3.3. The Necessity of Establishing Comprehensive Systems to Support Families

This category emphasizes the need for integrated systems to support families facing financial and social challenges. It stresses the importance of building strong networks between local supporters and organizations, collaborating with local governments, and ensuring effective communication with parents.

#### 3.3.1. The Need for Building Networks Between Supporters and Organizations

- “Community and child welfare officer are getting involved, and the conditions are improving” (M.).
- “It would be great to make better use of connections with food banks” (N.).
- “I hope we can share information with various organizations in the local area” (L.).

Creating networks between supporters and organizations is vital to strengthening the safety net for families. Effective collaboration among community members, child welfare officers, and local organizations is key to addressing challenges like poverty.

#### 3.3.2. The Critical Importance of Collaborating with Local Government for Effective Solutions

- “Solving the root causes of poverty isn’t something we can do as a community alone” (K.).
- “It’s also necessary to pass on the information we gather to government and related agencies” (P.).
- “Lawmakers and politicians need to create policies to ensure education and food security” (J.).

Collaboration with the local government is essential for addressing poverty’s root causes. While local communities play an important role, broader government support is needed to create policies that ensure education and food security.

## 4. Discussion

This study seeks to clarify the extent of child poverty and associated issues through FGD with stakeholders. Based on the findings, this discussion incorporates insights from previous research and provides a summary of child poverty and related issues in A City. In A City’s schools, some families struggled with expenses such as textbook fees, lunch fees, and event-related costs. The number of such families aligns with the “one in seven children in Japan lives in poverty” figure reported in UNICEF’s Innocenti Report Card 18 [1], highlighting that a significant number of households with children attending elementary and junior high schools in A City face economic hardship. Food insecurity and limited participation in extracurricular activities are recognized phenomena in high-income Asian societies, emerging within the context of children’s lived experiences in poverty [21]. Children often miss meals, indicating widespread food insecurity, a phenomenon that may also occur in Japan, a developed country, particularly in Tokyo, where higher-income individuals reside. In urban areas of Germany, children living in poverty sometimes arrive at school without breakfast or lack packed sandwiches for their break. Even when hungry, they might justify their lack of food by claiming to dislike the taste of the provided lunch or to not be hungry, attempting to conceal their poverty [22]. Such coping strategies suggest that food insecurity is not only a matter of physical deprivation but also a significant psychological burden, leading to feelings of shame, social isolation, and anxiety. Therefore, food security policies and interventions should be designed to minimize these psychological burdens while ensuring adequate nutritional support for children. Similarly, children from economically advantaged families may participate in supplementary educational activities outside regular school hours [23], potentially contributing to educational disparities. In South Korea, a similar dynamic exists, where the high cost of education, including private tutoring services, places a significant financial burden on families, exacerbating poverty [2]. The Tokyo Metropolitan Government has begun significant steps to reduce educational costs starting in fiscal 2024, including removing income caps on high school tuition subsidies and considering free school lunches for public elementary and junior high schools [24]. Although there is policy momentum to reduce educational costs, these findings suggest that challenges remain in A City, such as inadequate meals and exclusion from school events, underscoring the universal impact of poverty on education and daily life. Therefore, it is essential to establish systems in A City that enable teachers to identify and address student poverty at an early stage, as emphasized in the General Principles of Policy on Poverty among Children.

However, even when teachers notice signs of poverty, our study found that they often feel they “cannot easily contact the parents”. There are three main reasons for this. First, students and parents are reluctant to have their financial difficulties exposed to others. In the FGD, it was mentioned that “children do not speak up about their struggles”. Previous studies suggest that children living in poverty feel embarrassed and hurt and may fear discrimination from their peers [25]. Similarly, a study conducted in urban areas of Finland found that children from low-income families tend to conceal their economic situation [26]. They actively avoid situations where their family’s financial status might be revealed and refrain from identifying themselves as “poor”. Regardless of their actual financial circumstances, they tend to place themselves in the middle of the economic scale. This inclination could serve as a psychological mechanism to avoid self-stigmatization. Parents also often neglect their own basic needs to satisfy their children’s desires, fostering a relationship marked by affection, care, and loyalty [27]. In other words, the maintenance of the relationship between the child and their peers, as well as the emotions parents feel toward their children, may contribute to the hesitation in seeking support from others. Second, parents facing financial hardship might not recognize their situation as serious or in need of support, resulting in a lack of awareness of or access to available services. Third, there is a norm in Japanese schools that teachers cannot act until clear signs of distress or the need for help are evident from students [28]. However, as previous studies suggest, the school climate is crucial for students from low-income families, as a negative environment increases academic risks, while a positive one helps mitigate other stressors [29]. Therefore, it is essential to deepen our understanding of the psychosocial effects of poverty on students in the context of school management. In strengthening the quality of education, examples from the United States offer valuable insights that could inform Japan’s efforts. Two key areas in this regard are teacher preparation and school support. For example, residency programs recruit academically talented college graduates and support their work in high-poverty schools [30]. Additionally, organizations such as Teachscape and TeachingWorks focus on enhancing teacher preparation on a broader scale. Moreover, some states have successfully implemented comprehensive strategies to develop educators’ skills and improve underperforming schools [30]. These initiatives could represent a new approach for Japan, as schools, supported by both the government and private sectors, aim to improve the overall education system.

Our study revealed challenges in the surrounding community regarding support for struggling families. Modern Japanese society tends to emphasize avoiding being a burden on others rather than fostering mutual assistance. This perspective aligns with prior observations that modernization has led to a societal structure where individuals are encouraged to withdraw from personal relationships to avoid being perceived as a burden, making it more difficult for those in need to ask for help [31]. Parents of children living in poverty exhibited significantly lower levels of social capital, as indicated by reduced social trust, neighborhood cohesion, and mutual aid [32], suggesting weaker social connections and support networks compared to non-poverty households. A participant remarked, “Parents have pride and do not ask for help”, highlighting their reluctance to seek assistance due to concerns about burdening others or disclosing their struggles. This hesitancy to seek help may further reinforce the low levels of social capital observed among parents in poverty, creating a cycle of social isolation that restricts access to essential support systems. Furthermore, there is little opportunity to discuss stigma or prejudice in the context of poverty measures, both at the policy level and among the general public in Japan.

In our study, several challenges related to system-building emerged, particularly for local supporters such as social welfare organizations, in the context of future child poverty measures in A City. The first concerns the need to implement poverty programs that are integrated with municipal administrative efforts. Previous research on child poverty in Japan has consistently highlighted the importance of collaboration between the education and welfare sectors [33]. However, the network between these two sectors alone is insufficient. It is also necessary to build networks of both formal and informal community supporters, as these networks can play a crucial role in providing timely support. As pointed out in the FGD, the fundamental solution to child poverty cannot be achieved by the local community alone. A system must be established to share the needs identified in the field with the local government. It is believed that local governments in foreign countries also face significant challenges in combating child poverty, with the degree of success varying depending on governance structures, collaboration with local partners, and economic conditions [34,35,36]. On the other hand, as Azarnert LV points out, it is necessary to consider that humanitarian support provided to poor children on a per-child basis can entice parents to further reduce their own investment in their children’s education, thereby working against the goal of alleviating poverty [37].

The following points might be particularly significant in the context of children’s social environments. The points not addressed in the FGD of this study are twofold: material possessions and leisure activities. This is particularly relevant for children from low-income families, who often have a strong desire to conform to peer expectations through material possessions such as clothing and mobile phones [26]. These children might frequently feel different and excluded due to their inability to meet the fashion norms or consumer expectations of their peers. Similarly, regarding leisure activities, children from impoverished backgrounds are aware that they cannot participate in the same activities as their wealthier peers. As noted by participants in prior research, a child from a low-income family may only be able to play with friends in the neighborhood, while wealthier children can engage in activities such as roller skating, visiting amusement parks, or traveling abroad for vacations [38], as well as enjoying outdoor pools or attending summer camps [39]. This limited access to diverse leisure activities is suggested to lead to feelings of exclusion and boredom.

In this study, the opportunity provided by the FGD allowed stakeholders to share important insights regarding child poverty and related issues in A City. This is significant from the perspective of CBPR. In particular, it is important to highlight the contradiction. While the General Principles of Policy on Poverty among Children emphasizes the crucial role of community supporters, these supporters often find it difficult to intervene with children and families facing economic hardship in reality. Efforts to address child poverty centered around schools in A City are still in the developmental stages, and there is significant room for improvement in how these efforts are structured and implemented.

## 5. Conclusions

This study highlights the urgent need for an effective system to identify and address child poverty in A City. The findings emphasize the importance of fostering collaboration among schools, social welfare services, and community organizations to establish comprehensive support networks. The financial burden on families—including textbook fees, lunch fees, and event-related costs—has been documented in previous studies conducted in other countries [2,21,22,26], indicating that this issue is not unique to Japan. Furthermore, the finding that children frequently miss meals suggests widespread food insecurity, a phenomenon that might also be present in Japan, a developed country. This is particularly concerning in Tokyo, where higher-income individuals reside alongside families experiencing economic hardship. In response to these challenges, the Tokyo Metropolitan Government has recently taken significant steps to reduce educational costs, such as eliminating income caps on high school tuition subsidies and considering free school lunches for public elementary and junior high schools starting in fiscal 2024 [24]. While these measures represent progress, the challenges identified in this study persist in schools and local communities. In particular, teachers might hesitate to provide support due to concerns about maintaining children’s peer relationships, while parents may feel emotionally reluctant to seek assistance for their children, both of which could hinder effective intervention.

The reluctance to impose a burden on others [31] could be a culturally specific norm in Japan. Therefore, it is essential to deepen our understanding of the psychosocial effects of poverty on families experiencing economic hardship. In recent years, the number of Kodomo Shokudo (children’s cafeterias) has increased rapidly across Japan, with government support also playing a role in this expansion [40]. As a CBPR initiative, this movement could also play a key role in fostering networks among children, parents, and support providers in A City, contributing to the development of a more sustainable and inclusive support system.

## Figures and Tables

**Table 1:** Participants in the focus group discussion.

Name	Organization and Role	Contribution to Community and Schools
E.	Vice Principal of C Junior High School	Managing student discipline, curriculum implementation, and teacher support to maintain a structured learning environment. Collaborating with local organizations to provide career programs, mental health resources, and extracurricular activities to aid high school transition.
F.	Vice Principal of D Elementary School	Focusing on creating a safe, supportive environment for young learners, working with teachers and parents to promote early childhood education, behavioral development, and literacy programs through workshops and partnerships with child welfare organizations.
G.	Community and Child Welfare Officer	Coordinating welfare programs, child protection services, and community resources to support children and families in need. Collaborating with schools to identify at-risk students and develop initiatives promoting well-being, education, and inclusion.
H.	Community and Child Welfare Officer	Same as above.
I.	Member of A City’s food bank and a City Councilor of A City	Coordinating and leading food distribution efforts at the food bank to support vulnerable populations while overseeing day-to-day operations. Working as a city councilor to influence local governance, address community concerns, and promote initiatives that enhance social inclusion and well-being.
J.	A City’s food bank representative	Organizing and distributing food to those in need, coordinating resources effectively. Collaborating with local groups and advocating for community involvement and donations to sustain the food bank’s efforts.
K.	Member of A City Sports Club focused on youth development	Providing sports coaching and mentoring to youth, promoting physical fitness, teamwork, and personal growth. Leading programs that encourage healthy lifestyles and foster positive relationships among participants.
L.	Director of the elderly care facility operated by Corporation B	Overseeing operations and strategic direction of the facility, ensuring quality care and safety. Leading initiatives for healthcare services, staff training, and collaboration with local organizations to raise awareness on elderly care.
M.	Management staff member at the elderly care facility by Corporation B	Managing daily operations and resources to ensure efficient care for elderly residents, while creating a safe environment and collaborating with local health organizations and schools to educate the community on aging and health.
N.	Nutritionist at the elderly care facility by Corporation B	Developing nutrition programs for elderly residents, promoting health through balanced diets. Advocating for food security initiatives to ensure nutritious food access for all, including the elderly.
O.	Representative of the volunteers	Coordinating volunteer activities to support community members of all ages, from the youth to the elderly. Recruiting and training volunteers to provide companionship and engage in recreational activities.
P.	Board member of Corporation B	Providing strategic oversight and guidance to the elderly care facility, ensuring alignment with the organization’s mission and goals. Advocating for policies that enhance the quality of care for elderly residents and improve the facility’s services.

**Table 2:** Child poverty and associated issues in A City.

Category	Subcategory	Representative Quotes (Code Examples)	Participant
Children Disadvantaged by Their Family’s Financial Situation	Households unable to afford school-related costs	In a class of 30 to 35 students, about 5 of them have difficulty paying for school lunches.	E.
		There are households that are behind on payments for things like study materials and school lunches.	F.
	Students unable to participate in school events due to financial constraints	Some students couldn’t participate in school events because they didn’t have the money.	F.
		There are a certain number of families who find it hard to save up for the cost of the school trip.	E.
	Children who lack sufficient meals at home	I imagine that many children don’t eat breakfast before coming to school.	I.
		There was a student who depended on school meals for nutrition—it was a lifeline for them.	F.
Challenges in Supporting Financially Struggling Families in Schools and Communities	Difficulty in offering support to families struggling with financial hardship in schools	Many families aren’t struggling enough to qualify for financial support, like school aid.	G.
		Kids don’t speak up and say ‘I’m in trouble’ on their own.	E.
		Even if we notice that a student isn’t eating, it’s hard to contact the parents easily.	F.
		It’s difficult to understand a student’s family situation—it’s just not easy to find out.	O.
	Difficulty in gaining insight into families with children facing hardships in the community	In one visit to a single mother’s home, when we asked about their financial situation, they refused to answer.	G.
		I think parents don’t ask for help because of pride.	L.
		I imagine it’s hard for parents to accept free donations or assistance—there’s resistance to that.	J.
		Some families refuse to have child welfare services visit, and it’s hard to get involved with them.	H.
	Isolation of families due to the weakening of neighborhood communities	People just don’t care about what’s going on next door anymore.	K.
		The neighborhood association is weakening, and we don’t even know who our neighbors are.	H.
		With COVID-19, nuclear families have become more common, and now it’s more of a world centered on individual families.	J.
		The way parents live has a direct impact on their children’s environment.	I.
The Necessity of Establishing Comprehensive Systems to Support Families	The need for building networks between supporters and organizations	Community and child welfare officer are getting involved, and the conditions are improving.	M.
		It would be great to make better use of connections with food banks.	N.
		I hope we can share information with various organizations in the local area.	L.
	The critical importance of collaborating with local government for effective solutions	Solving the root causes of poverty isn’t something we can do as a community alone.	K.
		It’s also necessary to pass on the information we gather to government and related agencies.	P.
		Lawmakers and politicians need to create policies to ensure education and food security.	J.

## Data Availability

The original contributions presented in this study are included in the article. Further inquiries can be directed to the corresponding author.
